# Design and in vivo evaluation of a microparticulate depot formulation of buprenorphine for veterinary use

**DOI:** 10.1038/s41598-020-74230-6

**Published:** 2020-10-14

**Authors:** Viktoria Schreiner, Mattea Durst, Margarete Arras, Pascal Detampel, Paulin Jirkof, Jörg Huwyler

**Affiliations:** 1 Division of Pharmaceutical Technology, Department of Pharmaceutical Sciences, University of Basel, Klingelbergstrasse 50, 4056 Basel, Switzerland; 2 Center for Surgical Research, University Hospital Zurich, University Zurich, Zurich, Switzerland; 3 Department of Animal Welfare and 3Rs, University of Zurich, Winterthurerstrasse 190, 8057 Zurich, Switzerland

**Keywords:** Drug delivery, Pharmaceutics, Pharmacology

## Abstract

Buprenorphine is a frequently used analgetic agent in veterinary medicine. A major drawback, however, is the short duration of action requiring several daily administrations. We therefore designed a poly-lactic-co-glycolic acid (PLGA) based microparticulate drug formulation for sustained parenteral drug release. Particles were designed to allow for a fast onset of action and a duration of the analgesic effect of at least two days in laboratory mice. Microparticles were produced using a solvent evaporation technique. Release rate was dependent on polymer type and particle size. Spherical particles used for subsequent animal studies had a mean size of 50 µm and contained 4.5% of buprenorphine. Drug release was characterized by an initial burst release of 30% followed by complete release over seven days. In vivo pharmacokinetic experiments in female C57BL/6 J mice confirmed prolonged exposure in plasma and brain tissue and correlated with the pharmacological effect in the hot plate assay or after minor abdominal surgery. No adverse side effects with respect to food and water intake, body weight, local tolerability, or nesting behavior were observed. Our formulation is an attractive alternative to established immediate release formulations. A use for prolonged pain management in laboratory animals is proposed.

## Introduction

Buprenorphine is a semisynthetic opioid derivative frequently used for laboratory and companion animals^1,2^. Displaying several favorable characteristics like a 25–40 times higher potency than morphine in mice and rats^3^ and the occurring ceiling effect for respiratory depression^4–6^, buprenorphine is commonly used to alleviate pain after surgical interventions in rodents^7^. However, due to its short half-life of around 3 h in rodents^8–10^ repeated injections are necessary every 6 to 12 h^10,11^. The lack of available sustained-release buprenorphine formulations on the European market impede effective pain management and induce considerable stress on animals through the need of recurring injections and repeated animal handling^12^. Other opioids, which could be used as an alternative like morphine or fentanyl, fail to be as efficient as they are even more short acting^11,13,14^. Alternative routes of administration for buprenorphine, for instance the oral administration via drinking water, have also been discussed in recent years to reduce handling-associated stress. However, drinking behavior is dependent on the circadian rhythm^15,16^, thus making it impossible to ensure adequate buprenorphine levels for every mouse and every time point post-surgery resulting in unreliable pain alleviation. Nevertheless, sufficient pain-relief is required for animal welfare and reduces the risk for stress-induced artefacts^17^. Therefore, there is an urgent need to develop a biocompatible sustained-release formulation to prolong analgesic effect in laboratory animals.

In the last decades the biodegradable and safe polymer poly (lactic‐co‐glycolic acid) (PLGA) has been extensively studied and various carriers with different preparation procedures for sustained drug release were invented^18–22^. The use in humans has already been approved by the FDA for various products demonstrating the suitability of this polymer for in vivo use^23^. Several different types and compositions of PLGA are commercially available, enabling a tailor-made release over days or even months^18,20^. It should be noted, that a sustained-release buprenorphine formulation based on a different delivery strategy was recently approved by the FDA for the use in humans^24^.

Different sustained release products of buprenorphine appeared in the last years in the US for veterinary use. Buprenorphine SR-Lab® from ZooPharm (Fort Collins, CO) and Animalgesics for Mice® from Animalgesic Laboratories Inc. (Millersville, MD) are two FDA indexed formulations for experimental animals. Both formulations are depot formulations with a duration of action of at least 12–72 h in mice^25–27^ and 48–72 h in rats^28–30^. They are legally marketed unapproved animal drugs for minor species. As such, they are not commercially available outside of the US. It was therefore the aim of the current study to provide an alternative for the European market. These existing products are based on in situ forming implants or liquid suspensions and have certain limitations. This includes lesions at the injection site such as erythema and necrosis, suboptimal injection volumes, and viscosity leading to handling problems^25,29,31–39^. The novelty of our approach consists thus in the use of a lyophilisate with a proposed extended shelf-life, a reduced risk for injection site reactions, a lower dose and thus a reduced risk for side effects as compared to Animalgesics for Mice®, and a lower viscosity as compared to Buprenorphine SR-Lab® allowing for convenient handling.

Here, a novel size-controlled microparticulate depot formulation of buprenorphine based on the biodegradable polymer PLGA for prolonged and controlled pain reduction is proposed. Several different polymers were tested and characterized regarding size, morphology, drug load and in vitro release to obtain a formulation with roughly 30% burst release and a sustained release up to three days. Subsequent pharmacokinetic studies in naive, adult, female C57BL/6 J mice were done to assess plasma and brain exposure. Analgesic action for the novel depot formulation was compared to a non-retard buprenorphine formulation (Temgesic®) with a thermal sensitivity assay and Mouse Grimace Scale (MGS) scoring after sham-ovariectomy. Side effects and impairment of mice after surgery were monitored by nest building behavior and by clinical parameters like body weight, food, and water intake.

## Materials and methods

### Materials

Buprenorphine HCl (BUP) EP/USP grade was purchased from Macfarlan Smith Limited (Edinburgh, United Kingdom). Buprenorphine and buprenorphine-D_4_ (BUP-D4) standards in methanol (1 mg/mL) were obtained from Cerilliant (Round Rock, TX). Different types of PLGA 50:50 (Resomer RG 502, inherent viscosity (IV) = 0.20–0.22 dL/g), Resomer RG 502 H, IV = 0.20–0.22 dL/g, Resomer 503 H, IV = 0.38 dL/g) were purchased from Evonik (Essen, Germany). Polyvinylalcohol (PVA; M_w_ ≈67,000; 86.7–88.7 mol % hydrolysis), tris buffer and sucrose were obtained from Sigma Aldrich (St. Louis, MO). N,N-Dimethylformamide (analytical grade), dichloromethane (HPLC grade), methanol (HPLC grade), acetonitrile (MS grade), ammonium acetate (analytical grade) and sodiumlaurylsulfate (SDS) were obtained from Roth (Karlsruhe, Germany). HEPES was purchased from PanReac Apllichem (Darmstadt, Germany), sodium chloride (NaCl) analytical grade, formic acid and acetic acid (glacial) analytical grade from Merck (Darmstadt, Germany).

### Preparation of buprenorphine loaded PLGA microparticles

Loaded microparticles were prepared by an oil-in-water (O/W) emulsion—solvent evaporation technique. Buprenorphine HCl (5 mg) and PLGA (100 mg) were dissolved in 5 mL of dichloromethane. The organic solution was then slowly injected over a time period of 7.2 min into 75 mL of ice-cooled 1% (w/v) PVA aqueous solution containing 1 M NaCl and 25 mM tris buffer with a pH of 9. Emulsification was done using a three-bladed propeller stirrer (RW 16 basic, IKA-Werke, Staufen, Germany) at 600 or 1200 rpm for 15 min. The resulting emulsion was transferred to a baffled flask and subjected to overnight agitation to remove residual organic solvent. Solid particles were collected through centrifugation for 10 min at 21′000 g and washed three times with 50 mL Milli Q water to remove PVA and not encapsulated drug. The resulting microparticle pellet was reconstituted in Milli Q water and distributed in vials. Sucrose was added (3.3 mg/mL) and vials were lyophilized for 24 h. The lyophilization process was initiated by lowering the shelf temperature to -45 °C over a period of 1 h (linear temperature gradient) followed by a sintering step (shelf at -45 °C during 3 h). Primary drying was done at 0.011 mbar during 17.5 h. Temperature was raised from -45 to 10 °C during that time (linear temperature gradient). Secondary drying was done at 30 °C during 3.5 h at 0.001 mbar.

### Particle size and morphology of loaded microparticles

The size distribution of microparticles was measured by laser diffraction in water (MastersizerX, Malvern, Worcestershire, United Kingdom) using a lens suitable for particle sizes ranging from 1.2–600 µm. Particle size is expressed as the volume mean diameter and particle size distribution is expressed as ‘span’, which is defined by Eq. (1):

1 $$ span = \frac{{d\left( {0.9} \right) - d\left( {0.1} \right)}}{{d \left( {0.5} \right)}} , $$

where d(0.1), d(0.5) and d(0.9) are the particle sizes at which 10%, 50% and 90% of the sample are below this size.

To analyze morphology, lyophilized particles were mounted on double-sided adhesive carbon tape that was fixed on an aluminum stud and sputtered with 20 nm gold (EM ACE600, Leica, Wetzlar, Germany). Surface and inner structure of loaded microspheres was examined using focused ion beam scanning electron microscopy (FIB-SEM; Helios NanoLab 650, FEI, Hillsboro, OR) as demonstrated previously^40^. Briefly, a focused gallium beam was used to remove the top layers of polymer as well as to prepare cross-sections of particles, which were subsequent visualized by SEM.

### Measurement of drug loading and encapsulation efficiency

To measure drug content, lyophilized particles were dissolved in DMF, vortexed until a clear solution was obtained and analyzed by isocratic HPLC (Shimadzu Nexera X2 LC-30 AD, Kyoto, Japan). A reversed phase C_18_ column (Xbridge BEH, 4.6 mm × 50 m; 2.5 µm, Waters, Milford, MA) and a C_18_ guard column (SecurityGuard, 4.0 mm × 3.0 mm, Phenomenex, Torrance, CA) at 40 °C were utilized. A mobile phase of methanol:ammonium acetate buffer (20 g/L) :glacial acid (60:20:0.01) was delivered with a rate of 1.5 mL/min. BUP was detected at 288 nm. A stock solution of BUP in DMF was diluted with water to obtain a calibration curve ranging from 2 – 400 µg/mL.

Theoretical drug load (TDL) in percentage was calculated by Eq. (2):

2 $$ TDL = \frac{{m_{BUP} }}{{m_{BUP} + m_{PLGA} }}, $$

where m_BUP_ is the total amount of BUP (mg) and m_PLGA_ is the total amount of PLGA (mg) used for this formulation. Actual drug load (ADL) in percentage was defined as the ratio between measured amount of drug in the sample and total mass of the sample. Encapsulation efficiency (EE) in percentage was calculated as ratio between ADL and TDL.

### In vitro release study

To study the in vitro release, 60 mg of dried particles were dispersed in 7.5 mL 54 mM HEPES buffer containing 1.5% (w/v) SDS to ensure sink conditions. Samples of 300 µL for each time point in triplicate were incubated in closed glass vials at 37 °C on a horizontal shaker (250 rpm). To ensure homogenous mixing, vials were mounted on the shaker in a 45° tilted position. At specific time points, 260 µL of suspension was centrifuged for 8 min at 21′000 g and the supernatant was used for HPLC analysis to determine the cumulative release of BUP. Remaining particles were dissolved in DMF and analyzed by HPLC to obtain the total content of drug per vial in order to calculate normalized cumulative release. Burst release was defined as normalized BUP release after one hour.

### Animals

114 female 4 week old C57BL/6 J mice were purchased from Charles River Laboratories (Sulzfeld, Germany). Housing conditions and experimental procedures were approved by the Cantonal Veterinary Office Zurich and Basel, Switzerland, under the license 30,583, and were in accordance with the Swiss Animal Protection Law and also conform to European Directive 2010/63/EU of the European Parliament and of the Council on the Protection of Animals used for Scientific Purposes and to the Guide for the Care and Use of Laboratory Animals^41^.

A health surveillance program according to FELASA guidelines throughout the experiments monitored the animals’ health status. The mice were free of all viral, bacterial, and parasitic pathogens listed in FELASA recommendations^42^.

Animals were housed in groups of 2–8 animals in Eurotype III cages or in groups of 2–6 in Eurotype II Long cages (Techniplast, Hohenpeissenberg, Germany) with autoclaved sawdust bedding (LTE E-001 Abedd, Indulab, Gams, Switzerland), one tissue paper and two nestlets (5 × 5 cm, Indulab), one cardboard hut (Ketchum Manufacturing, Brockville, Canada) and/or one red plastic house (Techniplast, Hohenpeissenberg, Germany), one wooden enrichment tool (40 × 16 × 10 mm, Abedd, Vienna, Austria) and a wooden platform (235 × 125 × 12 mm, Abedd). Animals had ad libitum access to food (3430.PX.S15 12 mm, Granovit, Kaiseraugst, Switzerland) and sterilized drinking water. Room temperature was 21 °C ± 1, humidity 45% ± 2 and a light/dark cycle of 12 h/12 h (lights on at 8 am) was implemented. Animals in experiments that contained surgical procedures, were single housed for a period of 5 days. All animals were tunnel handled. The animal room was insulated to prevent electronic and other noise. Disturbances, e.g., unrelated experimental procedures in the animal room, were not allowed. Animals were sacrificed for sample collection. In order to reduce the total number of animals used, some mice were reused for other experiments (table S1). Detailed information about age and weight of used mice for different experimental set ups can be found in the supplement (table S1-4).

### Drug administration

Temgesic® (0.3 mg/mL buprenorphine HCl; Indivior Schweiz AG, Baar, Switzerland), a non-retard buprenorphine HCl formulation, was diluted with 0.9% NaCl (B.Braun, Hessen, Germany) to 0.02 mg/mL and given at a dose of 0.1 mg/kg body weight by subcutaneous injection in the lower belly region. Formulation RG 502 H-Big (BUP-Depot) was reconstituted with sterile saline solution to obtain a concentration of 240 µg/mL and injected subcutaneously with a dose of 1.2 mg/kg and a volume of 5 µl/g body weight once. Prior to injection, every batch of BUP-Depot was weighed and analyzed by HPLC regarding drug load.

### Pharmacokinetic studies

Animals were housed in groups of 2–4 in Eurotype II Long cages as described earlier. All experiments started at 8:00 a.m. in the morning with a subcutaneous injection of either non-retard formulation or BUP-Depot. Mice were randomly allocated by group and sampling time point. Detailed information about weight of animals and injected doses can be found in supplement (table S2). At specific time points (0.5, 2, 5, 12, 24, 48, 72 h) animals were anesthetized using isoflurane (Attane, Lyssach b. Burgdorf, Switzerland). Anesthesia was induced with 5% Isoflurane, maintained at 3%, and the heart was punctured with a syringe. Blood was collected, transferred to EDTA coated tubes (Microvette CB 300 K2E, Sarstedt, Nürmbrecht, Germany), and centrifuged at 3′000 g, 4 °C for 10 min. 50 µL of plasma was transferred to a 96 well plate and stored at -20 °C until further analysis. Directly after blood sampling, the entire brain was carefully extracted and dissected using a scalpel or razor blade into the following tissues: cerebellum, medulla oblongata with pons and remaining brain (midbrain and forebrain). All parts were subsequently weighed and stored at -20 °C. The specific binding of BUP in brain was calculated by subtracting drug concentration in cerebellum from combined concentrations in the remaining brain.

### Sample preparation and liquid chromatogram- mass spectrometry analysis

Buprenorphine plasma and brain concentrations were determined by LC–MS-MS using a Shimadzu Nexera X2 LC-30 system (Kyoto, Japan) coupled to a triple quadrupole mass spectrometer (Sciex QTRAP 6500, Framingham, MA). Deuterated buprenorphine (BUP-D4) was used as internal standard (IS). Calibration standards of buprenorphine covered a concentration range of 0.0032 to 50 ng/mL. Plasma (50 µl) and homogenized tissue samples were subjected to protein precipitation using acetonitrile, supernatants were collected, taken to dryness, and reconstituted with 100 µL of acetonitrile and 0.1% formic acid (4:6). For chromatographic analysis, two C_18_ columns were used (Sunshell 2.1 × 30 mm pre-column; 2.6 µm, ChromaNik Technologies, Osaka, Japan and Kinetex Biphenyl, 50 × 2 mm analytical column; 2.6 µm, Phenomenex, Torrance, CA). Gradient elution was used with dual-component mobile phase for both columns consisting of 0.1% (v/v) formic acid in water (Solvent A) and 0.1% (v/v) formic acid in acetonitrile (Solvent B) with a total flow rate of 0.45 mL/min. Column oven temperature was set to 60 °C and injection volume was 30 µL. Chromatographic separation was achieved by a gradient from 2% solvent B to 70% of solvent B over 6 min. Compound analysis was done in positive mode using a turbo spray temperature of 600 °C, an entrance potential of 10 V and an ion spray voltage of 4500 V. Detection of BUP and BUP-D4 was done using multiple reaction monitoring. Product ions with mass-to-charge ratios of 396.1, 414.2, 152.0, 165.1 were monitored for BUP and 400.1 (m/z) for BUP-D4.

### Pharmacodynamic studies

#### Analgesiometric assay

Animals were housed in Eurotype II long cages during the experiments and housing conditions of mice were the same as described above. Detailed information about weight of animals and injected doses can be found in supplement (table S3). At 8:00 a.m., equal numbers of mice were randomly assigned to receive subcutaneously, either one injection of BUP-Depot, non-retard formulation or saline solution with similar volume. The investigator performing the analgesiometric assay was blinded to treatment. Mice from each experimental group were tested at specific time points post injection (2, 12 or 24 h). Mice that received BUP-Depot were additionally tested 48 h post injection. Analgesic action was assessed by the hot-plate method^25,43^. Briefly, mice were habituated to the test room for 10 min and placed afterwards on a 54 ± 1 °C hot plate. Time was measured until mice showed one of the following behaviors: Hind-paw lick, hind-paw shake or jump. All experiments were either recorded by camera from two opposite sides of the hot plate or just from one side, while a mirror was placed on the other side to help detect the aforementioned behaviors. The investigator was watching the animals from a third side to remove animals from the plate after signs of nociception or a cut off time of 40 s. All latency measurements and evaluations were done blinded based on recorded video material.

### Assessment of pain relief and side effects after minor surgery

For the surgical procedure, 12 mice aged 14 weeks were housed individually for the experiment in Eurotype III cages with pre-weighed water and food. Detailed information about weight of animals and injected doses can be found in supplement (table S4). One fresh nestlet (5 cm × 5 cm), consisting of cotton fibres (Indulab AG, Gams, Switzerland) was given to each mouse to observe their nest building behavior before and after surgery. Two-times four animals per experiment were used, which were randomized to one of the two experimental groups: One injection BUP-Depot or two injections non-retard formulation. Experimental procedure was as follows:

- **Day 1:** At 8 a.m. individual housing started with a new nestlet and pre-weighed food and water. Animals were weighed for baseline measurement.
- **Day 2:** At 8 a.m. assessment of nest complexity score, monitoring of body weight, food and water intake was done. Baseline measurements for the Mouse Grimace Scale were conducted at 12 a.m., 2 p.m. and 9 p.m.
- **Day 3:** Prior to surgery at 8 a.m. assessment of nest complexity score, body weight, food and water intake was done.

### Surgery

Both groups received at 8:00 a.m. either non-retard formulation or BUP-Depot. Animals were transferred to the surgery room. At 9 a.m. anesthesia was induced via nose mask (5% isoflurane, 600 mL/min gas flow), animals were transferred to a warmed (39 ± 1 °C) operating table, and anesthesia was maintained (3% isoflurane, 600 mL/min gas flow) via nose mask. Eye ointment was applied, the fur was clipped and removed and the surgical site was disinfected with Braunol (B. Braun Medical AG, Sempach, Schweiz). Mice underwent a one-side sham embryo transfer as described previously^44^. Surgery was completed within 3–4 min in the surgery groups and total anesthesia time was 10 ± 1 min. While regaining consciousness after anesthesia, animals stayed for ∼10 min on the warmed table and were transferred afterwards to a warming cabinet (32 °C) for 60 min before returning to the housing room.

Mouse Grimace Scale measurements were done 3, 5- and 12 h post-surgery. Six hours post-surgery animals in the non-retard formulation group received their second injection with the same dose.

- **Day 4:** At 8 a.m., monitoring of nest complexity score, body weight, food and water intake after surgery.
- **Day 5:** At 8 a.m., monitoring of body weight, food and water intake after surgery.

### Nest building behavior, body weight, food and water intake

Nest complexity score was determined every morning at 8:00 a.m. prior to any intervention by a blinded observer using a scoring system (Score 0 -5) as described previously^44^.

Assessment of overall welfare was done through monitoring of body weight, food and water intake. Body weight of all animals as well as weight of food and water was measured every morning prior to any intervention.

### Mouse grimace scale

One day prior to surgery, baseline measurements of Mouse Grimace Scale were done as previously described^45^ at 12 a.m., 2 p.m., and 9 p.m. (corresponding to 3, 5 ,and 12 h after surgery). Briefly, mice were placed in Plexiglas cubicles (9 × 5 × 5 cm high) and filmed for 5 min. Frontal pictures of animals were generated and five were chosen for each mouse and time point and scored independently by two blinded observers for signs of pain. Pain scores were averaged for both observers. After surgery, the procedure was repeated at the same time points. Out of the five possible action units to code (orbital tightening, nose bulge, cheek bulge, ear position and whisker change), whisker change had to be excluded from analysis, as animals after surgery tended to stain the Plexiglas with eye ointment, making it difficult to examine the whiskers of mice properly. Values of individual animals were only included in results if three or more pictures could be completely scored. Delta Mouse Grimace Scale scores were obtained by subtracting the mean for the “after-surgery photographs” from the mean for the “baseline photographs” for each mouse and time point individually.

### Local-effects on injection site

Post injection of BUP-Depot and non-retard formulation, all mice were observed for local effects on injection site. If abnormalities like redness or swelling were detected, skin around injection site was removed post mortem and histologically analyzed.

### Statistical analysis

All statistics were done using OriginPro 2018 software (OriginLab Ltd, MA). Sample size calculation was based on previous experiments^43^ using the software GPower 3.1^46^. All data were tested for normality by performing a Shapiro–Wilk test. Analysis of variance (ANOVA) and two tailed t-tests were used. Level of significance was *P* < 0.05. For hotplate assay, ANOVA was combined with Tukey post hoc test. Baseline data for body weight, food and water intake were obtained by averaging measurements done on day 1 and 2 of experimental procedure. Scores were analyzed regarding significant differences between BUP-Depot and non-retard formulation group using non-parametric Mann–Whitney test.

## Results

### Characterization of buprenorphine loaded microparticles

Size, ADL, EE and burst release of different PLGA microparticle formulations are listed in Table 1. Burst release was defined as drug release after one hour and can be attributed to BUP encapsulated on or close to surface, easily accessible for hydration. The results showed that stirring speed had a major influence on particle size. Doubling the stirring speed with the polymer RG 502 H from 600 to 1200 rpm resulted in a decreased size of 11.7 µm compared to 49.8 µm, respectively. The same tendency could be seen with the higher molecular weight polymer RG 503 H, where particles produced with a higher stirring speed were only half the size of the particles produced with 600 rpm, namely 14.7 µm compared to 34.5 µm. Furthermore, particles with different sizes and same type of polymer showed differences regarding ADL, EE and burst release. Bigger particles of the polymer RG 502 resulted in an overall higher drug load (4.5% vs. 3.5%) and a higher EE (94.5% vs. 72.9%). Additionally, the larger particles displayed almost a doubling of the burst release (28.1%) compared to the smaller ones (15.7%). The same trend could be detected for the formulations prepared with RG 503 H. RG 502 H and RG 503 H have different molecular weights but very similar inherent viscosities of 0.22 dl/g and 0.38 dl/g. Particles have therefore similar size, ADL, and EE. However, burst release was slightly lower for smaller RG 503 H particles. Noticeable differences could be observed for particles formulated with the polymers RG 502 and RG 502 H. Both polymers consisted of similar chain lengths but differed regarding end capping. Particles formulated with these capped polymers using the same stirring speed of 600 rpm were only comparable in respect of size, but in terms of drug load, EE, and burst release they resembled more formulations with smaller particle sizes of the uncapped polymers (RG 502 H-Small or RG 503 H-Small). Here, ADL, and EE were almost identical, as well as burst release with 15.7% for RG 502 H-Small and 8.4% for RG 502-Big.

**Table 1:** Characteristics of sustained-release buprenorphine loaded microparticles. Data is presented as mean ± SD (n ≥ 3).

Formulation	Mw (kDa)	Endgroup	Stirring Speed (rpm)	Size (µm)	Span	Actual Drug Load (%)	Encapsulation Efficiency (%)	Burst Release (%)
RG 502 H-Small	13.4–15.1	–COOH	1200	11.7 ± 2.5	1.8 ± 0.1	3.5 ± 0.1	72.9 ± 2.0	15.7 ± 5.1
RG 502 H-Big* (BUP-Depot)	13.4–15.1	–COOH	600	49.8 ± 17.4	2.5 ± 0.4	4.5 ± 0.8	94.5 ± 12.9	28.1 ± 5.2
RG 503 H-Small	29.4	–COOH	1200	14.7 ± 4.2	1.7 ± 0.4	3.5 ± 0.1	72.7 ± 1.9	5.9 ± 2.4
RG 503 H-Big	29.4	–COOH	600	34.5 ± 10.3	1.5 ± 0.4	3.8 ± 0.5	82.1 ± 11.2	27.7 ± 10.6
RG 502-Big	13.3–13.4	–COOR	600	51.3 ± 22.5	2.6 ± 1.5	3.4 ± 0.3	71.4 ± 6.1	8.4 ± 5.8

*This formulation is the lead formulation used for all subsequent animal experiments. It is referred to as BUP-Depot.

### Effect of particle size and type of polymer on in vitro dissolution

**Figure 1:**
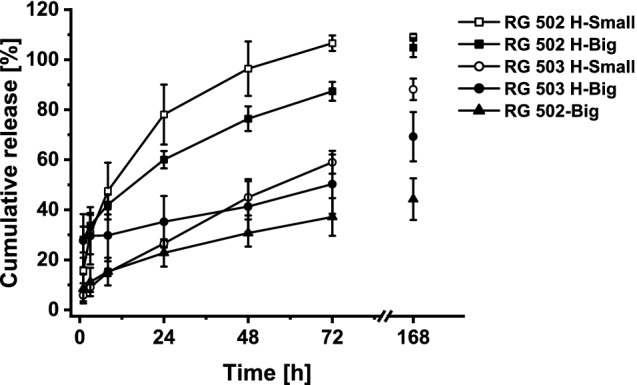
Effect of particle size and type of polymer on in vitro release of buprenorphine HCl loaded PLGA microspheres. Release was studied in 54 mM HEPES buffer at pH 7.4 with 1.5% SDS at 37 °C. Data is presented as mean ± SD (n ≥ 3).

In vitro release profiles of drug loaded microparticles in HEPES buffer (pH 7.4) incubated at 37 °C are shown in Fig. 1. Particle size and the type of polymer affected the in vitro release of buprenorphine from PLGA particles. Smaller particles in general displayed a faster release as could be seen in formulation RG 502 H-Small compared with RG 502 H-Big. Here, smaller particles reached 100% release after 72 h while the bigger particles attained only 87%, even though they had a higher burst release. RG 502 H-Big reached complete release between 3 and 7 days. A comparison of big and small particles prepared with the polymer RG 503 H showed the same trend. Hence, big and small particles reached almost the same release after 72 h of 50–59%, but the initial 20% higher burst release of the bigger particles meant that the release rate was much lower. Formulations with RG 503 H did not reach complete release within 7 days, but retained approximately 20–30% of drug. Release characteristics differed between polymers with higher and lower molecular weight. As expected, longer polymer chains demonstrated a slower release of the drug, as could be observed for RG 502 H-Small and RG 503 H-Small. Both formulations presented a similar particle size, but after 72 h only half (59%) of buprenorphine was being released from the higher molecular weight polymer compared to a complete release from the lower molecular weight version. Additionally, formulations with RG 503 H presented an almost zero order release after initial burst phase for the duration of 72 h. In contrast, the release profile of formulations with RG 502 H showed a power law release kinetic. This study showed furthermore that acidic end groups of polymers like RG 502 H and RG 503 H had an increasing effect on BUP release in vitro. Particles prepared with RG 502 H resulted in a complete drug release after 3 days, while particles with the same size formulated with capped polymer RG 502 released only 37%. Formulations with this type of polymer followed a power law release kinetic similar to RG 502 H. Formulation RG 502 H-Big, with a complete drug release of 3 days combined with a burst release of around 30%, was further characterized and used for all subsequent in vivo studies.

### Morphology and inner structure of loaded particles

**Figure 2:**
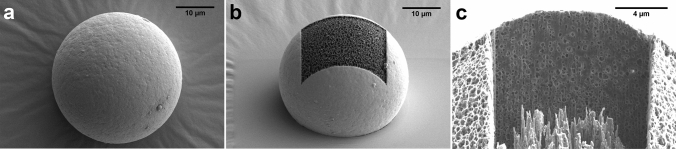
Scanning electron microscopy pictures of buprenorphine loaded PLGA (RG 502 H) microspheres**.** Focused ion beam technique was used to examine inner structure through removal of top layer of polymer (**b**) and vertical sectioning of particles (**c**).

Microparticle morphology was studied using scanning electron microscopy (Fig. 2a). BUP loaded particles formulated with the polymer RG 502 H showed a uniform, spherical shape and a smooth outer surface. In contrast, the inner structure of the spheres, revealed through ablation by a focused ion beam, was highly porous (Fig. 2b, c). Pores were homogeneously distributed throughout particles as can be seen from the vertical sectioning image in Fig. 2c.

### Pharmacokinetics

**Figure 3:**
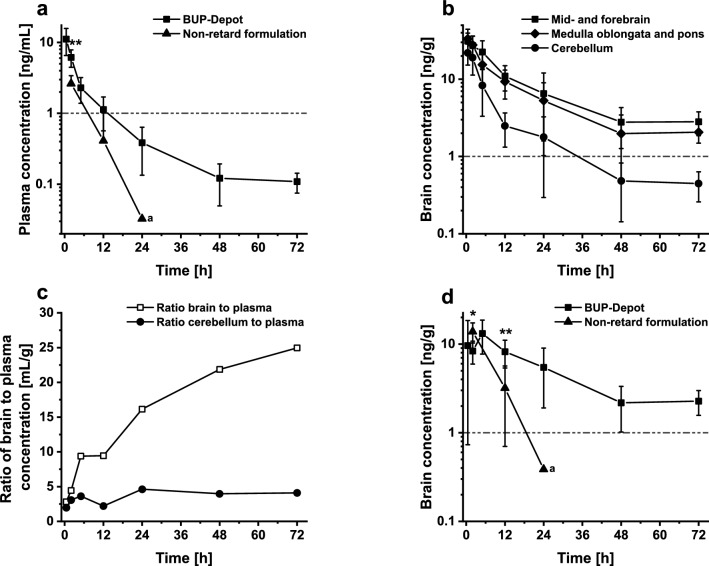
Plasma and regional brain concentration–time profiles. (**a**) Plasma concentration–time profile of RG 502 H-Big (BUP-Depot) and non-retard formulation. Unpaired t-test showed significant difference between formulations for 2 h (*P* = 0.0009) but not for 12 h (*P* = 0.051). (**b**) Regional brain concentrations of BUP-Depot in mid-and forebrain, medulla oblongata and pons, and cerebellum. (**c**) Brain-to-plasma ratio-time profiles of BUP-Depot. Brain concentrations are defined as sum of concentrations of mid- and forebrain, medulla oblongata and pons. (**d**) Specific binding of BUP-Depot and non-retard formulation in brain. Specific binding is defined as difference between combined concentrations of mid- and forebrain, medulla oblongata and pons and concentrations in cerebellum. Unpaired t-test showed significant difference at 2 h (*P* = 0.01) and 12 h post injection (*P* = 0.006) between both formulations. BUP-Depot (1.2 mg/kg) and non-retard formulation (0.1 mg/kg) were injected once subcutaneously. Dashed line in all graphs represents threshold of 1 ng/mL or 1 ng/g. Data expressed as mean ± SD. a: 24 h time point of non-retard formulation could not be statistically analyzed as only 2 out of 7 animals showed values higher than LLOQ for concentrations in plasma and cerebellum. Values below LLOQ were excluded, therefore no SD value is shown. (*P* < 0.05 *; *P* < 0.01 **).

Analytes were measured by LC–MS-MS in positive mode with a LLOQ of 0.008 ng/mL for plasma and 0.048 ng/mL for brain tissue. Single injection of BUP-Depot led to a biphasic release profile in mouse plasma as indicated in Fig. 3a. The first part of the profile could be attributed to the burst release of the retard formulation until 5 h post injection. Following the initial fast and immediate release of BUP, a slower sustained release phase could be observed until 72 h. At 12 h post injection, plasma concentrations started to approximate the mark of 1 ng/mL with a concentration of 1.1 ng/mL. Thereafter, plasma levels decreased further until a value of roughly 0.1 ng/mL was reached for the duration of 72 h. In contrast, a single injection of non-retard BUP formulation presented a profile with only one phase, where plasma concentrations above 1 ng/mL could only be measured up to two hours post injection. After 12 h plasma concentration dropped to 0.4 ng/mL but no significant difference to BUP-Depot could be established (*t* (11) = 2.20, *P* = 0.051). At 24 h post injection only 2 out of 7 mice showed detectable drug concentrations, whereas all other values were below the limit of quantification and were not included. Therefore, no statistical analysis could be performed.

In addition to plasma, brain tissue as the target site of BUP was analyzed. Figure 3b shows BUP concentrations in different parts of mouse brain after a single injection of BUP-Depot. In both tissue fractions, (medulla oblongata with pons and mid-/forebrain) concentrations above 1 ng/g for the whole duration of 72 h were observed. While medulla oblongata and pons and mid-and forebrain displayed similar concentrations of drug over 3 days, the amount of BUP in the cerebellum was much lower. Same trend can be seen in mice receiving non-retard formulation (Supplement, figure S1). This difference could be attributed to differential expression of opioid receptors in the brain, i.e. absence of receptors in cerebellum which was therefore used as baseline control tissue^47^.

Consequently, the ratio of cerebellum to plasma concentrations represents unspecific perfusion and does not change. In contrast, brain (i.e. mid-/forebrain combined with medulla oblongata and pons) to plasma concentrations increased over time by a factor of eight being indicative of specific receptor binding and tissue accumulation (Fig. 3c).

Based on considerations above, ‘specific binding’ of drug in the brain was defined as difference between combined concentrations of mid- and forebrain, medulla oblongata and pons and concentrations in cerebellum (Fig. 3d). In all subsequent experiments, the term ‘specific binding’ refers to this differential value. The maximum reached specific binding concentration of BUP was similar for the non-retarded and the depot formulation, 13.8 ng/g and 13.2 ng/g, respectively. However, the time until the maximum concentration of drug was reached was increased for the BUP-Depot formulation with 5 h compared to the non-retard formulation with 2 h. At 12 h post injection concentrations achieved through depot formulation were significantly higher compared to non-retard solution (*P* = 0.006). At 24 h post injection the specific binding concentration for BUP-Depot was still above 1 ng/g with 5.4 ng/g, whereas mice receiving the non-retarded formulation experienced a much lower value, 0.4 ng/g. Here the concentration of BUP in cerebellum could not be detected for 5 out of 7 mice, as concentrations dropped below LLOQ and were not included in the analysis. In contrast, up until the last measurement at 72 h post injection BUP-Depot still demonstrated concentrations above 1 ng/g with a value of 2.3 ng/mL. Only one mouse showed drug concentrations below LLOQ.

### Pharmacodynamics

#### Analgesic efficacy of BUP-Depot compared to non-retard formulation

**Figure 4:**
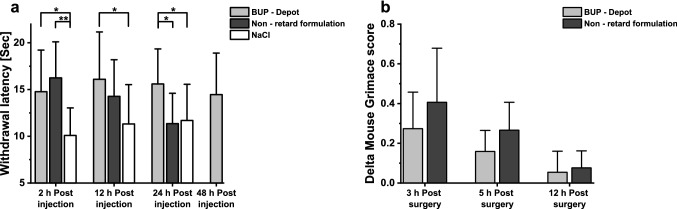
Effect of sustained-release buprenorphine (BUP-Depot), non-retard formulation and saline on withdrawal response latency to thermal stimulus of 54 °C in mice and on delta mouse grimace score after sham-ovariectomy. (**a**) One way analysis of variance with post hoc Tukey test shows significant (*P < 0.05;**P < 0.01) increase in latency for BUP-Depot at 2 h, 12 h, 24 h time points compared to NaCl and compared to non-retard formulation at 24 h. Non-retard formulation shows only a significant increase in latency at the two hours’ time point compared to NaCl animals. (n ≥ 11 per time point and group). (**b**) Standard analgesic protocol was used after sham-ovariectomy, treatment groups received either one injection of BUP-Depot or two injections of non-retard formulation 6 h apart. No significant difference between delta mouse grimace scores could be shown for both groups 3–12 h post-surgery using Mann–Whitney test (α = 0.05). Data expressed as mean ± SD for all experiments. (n = 6 per group).

Withdrawal latencies in response to heat stimulus of 54 ± 1 °C were measured after a single injection of either BUP-Depot, non-retarded BUP, or NaCl. Results of hotplate assay are presented in Fig. 4a. First testing was performed two hours post injection, as non-retarded BUP should display a maximum effect at that time, which served as a positive control. Further experiments were conducted 12 and 24 h post injection for all three treatment groups and 48 h only for BUP-Depot, since it was the only group where a pain reduction was still conceivable. Analysis of variance followed by post hoc pairwise comparison of means with Tukey correction revealed that 2 h post injection withdrawal latencies were significantly higher for BUP-Depot (*P* = 0.013) and non-retard formulation (*P* = 0.001) compared to control group which received NaCl. Furthermore, 12 h post injection mice receiving sustained-release BUP displayed significantly higher latencies compared to NaCl control animals (*P* = 0.03). No significant difference was found between latencies for non-retard formulation compared to NaCl (*P* = 0.24), confirming the short duration of action of the commercial formulation. Tested again 24 h post injection, withdrawal latencies for BUP-Depot were still significantly higher compared to baseline (*P* = 0.038) and to non-retard formulation (*P* = 0.02). Mice receiving commercial non-retard formulation displayed comparable latencies as NaCl animals (*P* = 0.97), which is in line with the pharmacokinetic data which showed that 24 h post injection BUP concentrations in plasma or brain were very low. The latest analgesiometric test was performed 48 h post injection only with a BUP-Depot group as latencies of control animals receiving NaCl did not vary much between different time points and no effect of non-retard formulation could be expected. Withdrawal latencies for animals receiving BUP-Depot were still higher at 48 h post injection with 14.5 s compared to baseline animals measured 24 h post injection with 11.7 s, but unpaired t-test showed that difference was not significant (t(21) = 1.59, *P* = 0.13).

Analgesic action of BUP-Depot compared to non-retard formulation was further assessed after surgery with standard analgesic protocol to alleviate pain. Mouse Grimace Scale was used to determine pain of animals 3, 5 and 12 h after surgery (Fig. 4b). Although a tendency towards lower delta MGS scores indicating lower pain was seen at 3 and 5 h, no significant difference between treatment groups for all time points could be shown (3 h: U = 11.5, *P* = 0.34; 5 h: U = 9, *P* = 0.17; 12 h: U = 19; *P* = 0.94).

### Correlation between drug concentration in plasma or brain and analgesic effect

Figure 3a and 3d present drug concentrations of BUP-Depot in plasma as well as in the brain. Both profiles showed a different distribution of buprenorphine over the course of 72 h. While plasma concentrations fell below 1 ng/mL after 24 h, the drug concentration in the brain remained above 1 ng/g for the whole duration of 72 h. Combined with the analgesic effect shown in Fig. 4a, a better correlation between drug-brain-concentrations and analgesic action could be shown compared to the plasma concentration.

### Effect of BUP-Depot on nest building behavior, body weight and food and water intake after surgery compared to non-retard formulation

**Figure 5:**
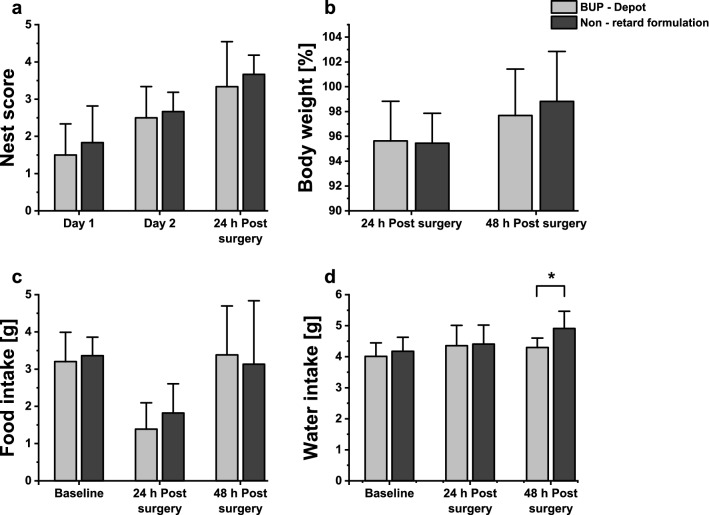
Effects of one injection of BUP-Depot compared to two injections of non-retard formulation after sham-ovariectomy on nest complexity score, body weight, food and water intake. (**a**) Nest scores during baseline measurements on day 1 and 2 and 24 h post-surgery show no significant differences between both groups at any time point (Day 1: *P* = 0.92; Day 2: *P* = 0.73; 24 h: *P* = 0.92). (**b**) Body weight change in percent 24 and 48 h post-surgery show no significant difference between BUP-Depot and non-retard formulation using unpaired two-tailed Student t-test (24 h: *P* = 0.91; 48 h: *P* = 0.62). Food intake (**c**) and water intake (**d**) in gram during baseline measurements, 24, and 48 h post-surgery. Baseline measurements consists of average data from day 1 and day 2 of procedure. No significant difference between non-retard formulation and BUP-Depot group could be found using unpaired two-tailed Student t-test ((**c**) Baseline: *P* = 0.69; 24 h: *P* = 0.34; 48 h: *P* = 0.78), (**d**) Baseline: *P* = 0.54; 24 h: *P* = 0.88). Only exception was 48 h post-surgery water intake, indicating significant difference between BUP-Depot and non-retard formulation group (*P* = 0.038). Data is expressed as mean ± SD. (n = 6 per group).

The goal was to show similar effect of non-retard and sustained release formulation on mice after surgical procedure. Therefore, no difference was expected between both groups. Reduced nest building behavior is used as indicator of distress after surgical interventions in mice^44,48^. Analysis of nest scores of animals receiving either one injection BUP-Depot or two injections of non-retard formulation after sham-ovariectomy are presented in Fig. 5a. Regardless of the received formulation, mice built and improved their nests. Both groups had similar scores of 3.3 and 3.7 for BUP-Depot and non-retard BUP treated animals 24 h post-surgery, respectively. No significant difference could be found 24 h post-surgery between both groups (U = 17, *P* = 0.92). Body weight change (Fig. 5b) was not significantly different between non-retard formulation and BUP-Depot group for all time points (24 h: t(10) = 0.12, *P* = 0.91; 48 h: t(10) = -0.51, *P* = 0.62). Mice lost around 4% of their body weight after surgical intervention 24 h post-surgery. Already 48 h after surgical intervention, body weights of both groups (97.7% for BUP-Depot and 98.8% for non-retard solution) were similar compared to baseline, hinting that both groups recovered fast. Similar to body weight change, food intake was not significantly different if Depot and non-retard group were compared before or after surgery (Fig. 5c) (Baseline: t(10) = -0.41, *P* = 0.69; 24 h: t(10) = -1.01, *P* = 0.34, 48 h: t(10) = 0.28, *P* = 0.78). Mice ate less 24 h post-surgery, around 1.4 g of pellets for BUP-Depot and 1.8 g for non-retard formulation. Food intake for both groups was again similar to baseline values 48 h after the surgical procedure, indicating good recovery. While no significant difference could be found 24 h post-surgery between water intake for mice that received BUP-Depot or non-retard BUP (t(10) = -0.15, *P* = 0.88), after 48 h post-surgery animals that received two injections of non-retard formulation showed a significant increase in water intake (Fig. 5d, t(10) = -2.38, *P* = 0.038).

### Local-effects on injection site

Only two mice out of 75 receiving the BUP-Depot formulation demonstrated a local reaction after 48 h, which manifested as mild skin irritation and swelling. Post-mortem analyses revealed one animal with a mild inflammation and another animal with a focal area of necrosis at injection site (data not shown).

## Discussion

Animal welfare is of paramount importance in biomedical research and includes the critical analysis of existing pain management protocols. One of the most commonly used analgesics to relieve moderate to severe pain in rodents is the opioid buprenorphine. However, its duration of action of only 6 -12 h requires repeated injections after most surgical interventions^11^.

In the present study, the biodegradable and biocompatible polymer poly (lactic-co-glycolic acid) was chosen to achieve prolonged drug release. PLGA is typically utilized to attain sustained-release over the course of several weeks (i.e. Lupron Depot, Eligard, Nutropin Depot and Profact). Buprenorphine is commonly administered for post-surgical pain after moderate to major surgeries for up to three days. Hence, ratios of 50:50 polylactic acid to polyglycolic acid were used in this study, as this composition is described to be fast releasing compared to bigger shares of PLA^49,50^. Furthermore, the molecular weight of the elected polymers were on the lower range, as smaller polymer chains degrade faster and hydration is accelerated due to a higher number of carboxylic acid end groups^51–53^. This approach was supported by the observation of a nearly complete in vitro release of 87% for RG 502 H-Big which was achieved within three days, whereas only 69% drug was released for RG 503 H-Big after one week. It is interesting to note that larger particles showed a higher burst release despite their smaller specific surface area. It is tempting to speculate that buprenorphine is not evenly distributed throughout particles and that bigger particles might therefore have higher surface bound drug concentrations. However, exploratory results using energy-dispersive X-ray microanalysis failed to provide distinct distribution patterns of drug within particles due to melting of PLGA during irradiation. Other polymers regularly used for sustained-release formulations like PCL or PLA were not considered for this work due to their unfavorable and slow release characteristics.

A burst release can be attributed to drug solubilization from or close to the surface of the carrier. As this is often regarded as unfavorable in achieving a consistent and continuous release profile^54^, a burst release of around 30% was favorable in the present study, to ensure full analgesic effect immediately after administration. With a burst and cumulative release over three days of 28% and 87%, respectively, formulation RG 502 H-Big was most suitable for all further studies. An alternative formulation for use in vivo could be RG 502 H-Small with a burst release of 16% and complete in vitro release between 48 and 72 h. In such a scenario, due to the lower burst release, an additional loading dose would be needed. However, such an approach would complicate handling and formulation preparation. Therefore, formulation RG 502 H-Big was used for all in vivo studies.

The amount of BUP for the depot formulation was chosen based on the standard therapeutic dose for mice, which is 0.1 mg/kg every 6 h. Hence, 12 injections are necessary resulting in a total dose of 1.2 mg/kg for three days. Accordingly, a total dose of 1.2 mg/kg BUP of the sustained-release formulation was administered to cover the same period. Pharmacokinetics of the depot formulation were assessed through plasma-and brain-concentration–time profiles and compared to non-retard BUP. Previous studies have shown therapeutic effective concentrations of BUP in plasma with a threshold of around 1 ng/mL for rodents^55,56^. In the present study, a single injection of non-retarded BUP resulted in plasma concentrations above 1 ng/mL only at the first measured time point at 2 h. At 12 h plasma levels declined considerable (0.4 ng/mL) and are almost not detectable at 24 h, since 5 out of 7 mice showed values below the LLOQ. The presented findings correlate with previous studies, indicating that a non-retard dose of 0.1 mg/kg results in high enough BUP plasma concentrations for a couple of hours only^43,57,58^. The described burst release of the depot formulation contributed to the fast rise and decline of drug levels in the first hours, which represented the first phase of the pharmacokinetic profile. Thereafter, the sustained-release formulation showed a second phase, including plasma levels close to 1 ng/mL for the duration of 12 h and a detectable concentration of drug until the last measurement point at 72 h, demonstrated that a sustained drug release was achieved.

While the threshold of 1 ng/mL is often referenced for assessing analgesic action of buprenorphine, this only takes plasma or blood levels into account^55,56^. As an opioid derivative, buprenorphine executes its analgesic effect through various opioid (µ-/κ-/δ-) receptors in the brain^59,60^. Ohtani et al. have shown that a good correlation exists between specific binding concentrations of BUP in the brain and analgesic effects in rats. Furthermore, analgesic activity and plasma concentrations did not correlate, especially in the early phase after administration^61^. Therefore, brain exposure of BUP over time was analyzed and correlated to the analgesic effect shown in the thermal sensitivity assay. Contrary to the plasma profiles, which dropped below 1 ng/ml, a single injection of the sustained-release formulation lead to an elevated specific binding concentration in the brain of 2.3 ng/g until the last measurement point at 72 h. While after injection of the non-retarded formulation the specific binding concentration of BUP in the brain showed a higher exposure compared to plasma level after 12 h, the concentration in the brain decreased significantly thereafter. Taking the results from the thermal sensitivity assay into account, specific binding concentrations of BUP in the brain of 3 ng/g might not be enough to alleviate pain reliably, as can be seen for mice receiving non-retard solution after 12 h (Fig. 4a). Withdrawal latencies at that time point show still higher values with 14.3 s for non-retard formulation compared to 11.3 s for control animals but no significant difference can be found. In contrast to that, animals receiving depot formulation showed significantly increased withdrawal latencies even 24 h post injection, which correlates to drug concentrations of around 5 ng/g in brain. It could be speculated that specific binding concentration values of 5 ng/g in the brain need to be achieved to reliably relieve pain in mice. Therefore, it could be expected that one injection of depot formulation alleviates strong pain only up to 24 h. Withdrawal latencies measured 48 h post injection showed still high values but no significant differences could be established to control animals at the 24 h time point , which correlates to specific binding concentrations of less than 3 ng/g at 48 and 72 h post injection. Analgesic performance determined through thermal sensitivity assay confirmed the short duration of action of the non-retarded formulation shown previously^11,16,43^. In line with plasma and brain concentration profiles, one injection of the non-retarded solution resulted only at the first 2 h time point in significantly higher withdrawal latencies (Fig. 4).

Buprenorphine is a potent analgesic drug and is therefore used to alleviate moderate to severe pain caused by e.g. surgeries^48^. Consequently, effectiveness and adverse effects have to be judged after clinically relevant, i.e. surgical, pain stimuli. In the present study, a sham ovariectomy was performed, which caused mild to moderate pain for less than 24 h^48,62^. One subcutaneous injection of sustained-release formulation was compared to two injections of a non-retarded BUP solution. Results confirmed the findings of the hot plate assay.

Since the maximal plasma and brain levels for both formulations were comparable, no difference in systemic side effects like body weight reduction, decreased food and water intake, and nesting behavior^44,48,63–65^ were anticipated. In particular, decrease in food and water intake, and thereof reduction of body weight, are widely described after surgeries but are also well-known side effects of opioids^16,63,66,67^. Both formulations led to a decreased food intake and body weight 24 h after surgery but did not differ from each other. Other studies have shown, that increased handling stress due to recurring injections can lead to a further decrease in body weight or food intake^43^. Since animals treated with the non-retarded solution received only two injections, handling stress was only minimal and therefore not expected to result in differences between the protocols. No further adverse events have been noted regarding sustained-release formulation, except two animals with local effects at injection site as described above. Nevertheless, further studies have to be conducted to confirm the safety profile of the depot formulation.

Buprenorphine is a well-established drug and has been on the market for decades, demonstrating safety and efficacy^68^. However, pain is not only a highly inter-individual variable, but recent research demonstrates that pain mediation differs depending on mouse strain and sex^69,70^. For the present study, only female C57BL/6 J mice were used. It remains to be elucidated if buprenorphine doses have to be adjusted for other mouse strains, rats, or males.

In conclusion, a novel depot formulation of buprenorphine based on biodegradable PLGA microparticles was successfully developed. Specific binding concentrations of BUP in the brain as the target site of opioids show therapeutic levels of drug for at least 24 h and a trend towards pain relief for at least 72 h. Thermal sensitivity assay confirms analgesic effect for at least 24 h, which is a significantly prolonged duration of action compared to the marketed standard formulation. In a proof-of-concept, post-surgical pain relief was accomplished in sham ovariectomy without notable side effects. The presented buprenorphine depot formulation offers a more efficient and less stressful pain relief for laboratory mice and can therefore be considered a refinement of current analgesic protocols. A possible use of the new buprenorphine depot formulation for companion animals, such as cats and dogs, would considerably widen the field of application.

## Supplementary information

Supplementary file1

## Data Availability

The authors confirm that the data supporting the findings of this study are available within the article and its supplementary materials.
