# Supplementary material for: Design and in vivo evaluation of a microparticulate depot formulation of buprenorphine for veterinary use
Source: Sci Rep. 2020 Oct 14;10:17295. doi: 10.1038/s41598-020-74230-6 (PMC7560740; doi:10.1038/s41598-020-74230-6)
Supplement: Supplementary file 1 — Supplementary file1 [file 41598_2020_74230_MOESM1_ESM.pdf]

Design and In Vivo Evaluation of a Microparticulate Depot Formulation of Buprenorphine for Veterinary Use

Viktoria Schreiner[1], Mattea Durst[2], Margarete Arras[2], Pascal Detampel[1], Paulin Jirkof[2,3]*, Jörg Huwyler[1]*

1 *Division of Pharmaceutical Technology, Department of Pharmaceutical Sciences, University of Basel, Basel, Switzerland*

2 *Center for Surgical Research, University Hospital Zurich, University Zurich, Zurich, Switzerland*

3 *Department of Animal Welfare and 3Rs, University Zurich, Zurich, Switzerland*

Supplement

Table S1: ***Number of mice used for different experimental set ups.***

**1. Mice used for hotplate assay and for PK study (Mice were only used once)**	
**Group**	**Number of Mice**
BUP-Depot	24
Non-retard Formulation	18
NaCl	0
**2. Mice used for PK only**	
**Group**	**Number of Mice**
BUP-Depot	21
Non-retard Formulation	2
NaCl	2
**3. Mice used only for hotplate assay once**	
**Group**	**Number of Mice**
BUP-Depot	15
Non-retard Formulation	0
NaCl	1
**4. Mice used for hotplate assay twice [a]**	
**Group**	**Number of Mice**
First Round	
Non-retard Formulation	22
NaCl	9
Second Round	
BUP-Depot	9
Non-retard Formulation	9[b]
NaCl	13[b]
**5. Surgical procedure [b]**	
**Group**	**Number of Mice**
BUP-Depot	6
Non-retard Formulation	6

[a] A total of 31 mice were used twice for the hotplate assay. After first round, mice were allocated randomly to new groups after a wash-out phase of 6 days to a second round. [b] Mice that were used twice for the hotplate assay and received only non–retard formulation or NaCl were used after a wash-out phase of 41 days for surgical procedures.

Table S2: ***Number of mice per group with average weight and average applied dose used for pharmacokinetic study.*** *Data is presented as ± SD.*

Group	Number of Mice	Time Points	Number of Mice per Time Point	Weight [g]	Dose [mg/kg]
**BUP-Depot**	45	0.5, 2, 5, 72 hours	6	17.7 ± 1.4	1.1 ± 0.04
		12, 24, 48 hours	7		
**Non-retard Formulation**	22	2 hours	6	17.2 ± 1.9	0.12 ± 0.01
		12, 24 hours	7		
**NaCl**	2	-	-	19.0	-

Table S3: ***Number of mice per group and time point with average weight and average applied dose used for hotplate assay.*** *Data presented as ± SD.*

Group	Number of Mice per Time Point	Time Points	Weight [g]	Dose [mg/kg]
**BUP-Depot**	12	2, 12, 48 hours	18.3 ± 1.3	1.1 ± 0.1
	11	24 hours		
**Non-retard Formulation**	12	2, 12, 24 hours	18.1 ± 1.8	0.11 ± 0.01
**NaCl**	12	2, 12, 24 hours	18.8 ± 1.8	-

Table S4: ***Number of mice per group with average weight and average administered dose used for surgical procedure (sham ovariectomy).*** *Data presented as ± SD.*

Group	Number of Mice	Weight [g]	Dose [mg/kg]
**BUP-Depot**	6	21.8 ± 1.3	1.1 ± 0.1
**Non-retard Formulation**	6	22.2 ± 1.2	0.11 ± 0.002

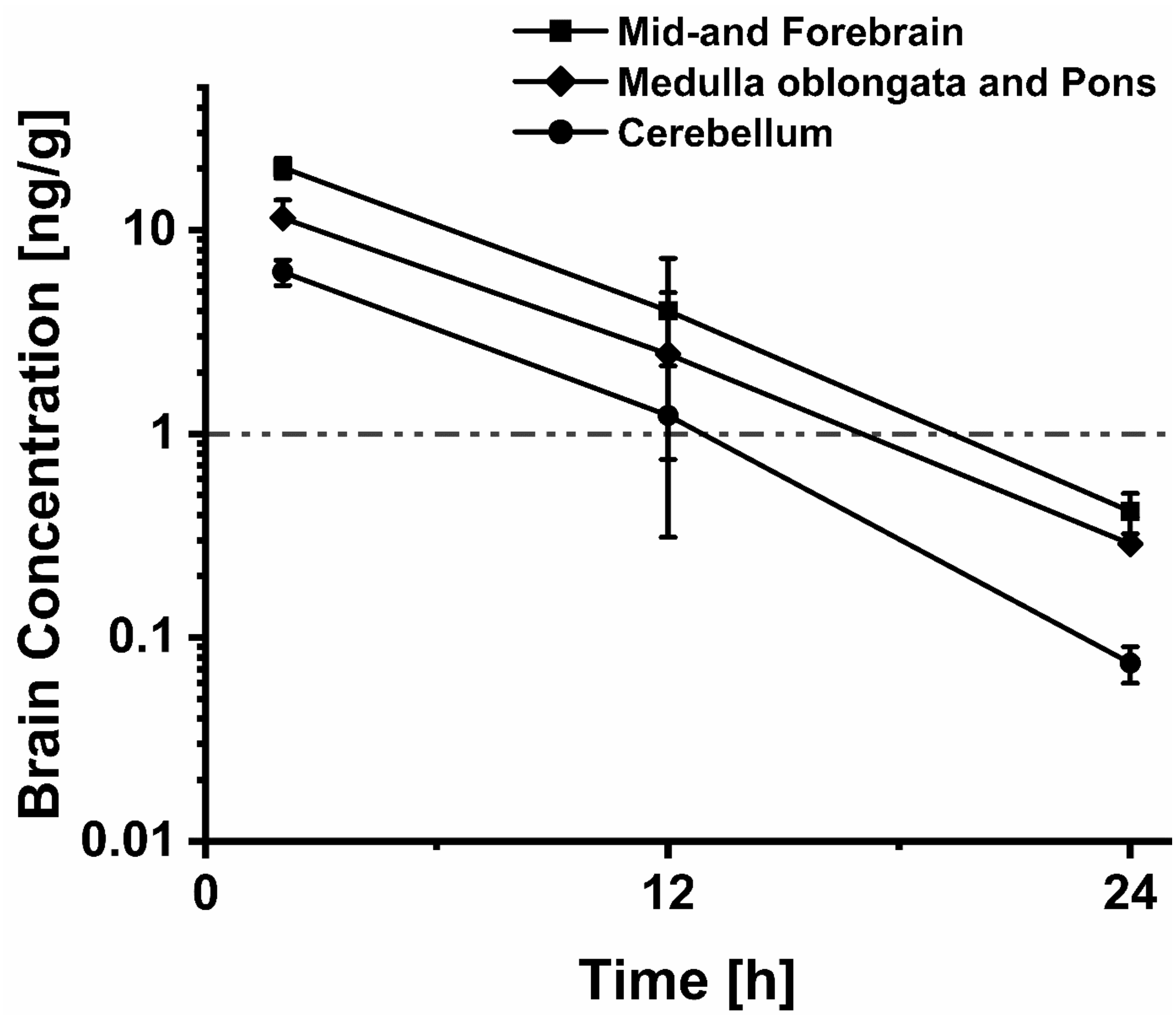

Figure S1: ***Regional brain concentration-time profile of non-retard formulation after single subcutaneous injection (0.1 mg/kg)****. Dashed line represents 1 ng/g and data is expressed as mean ± SD.*
